# Evaluating the quality and reliability of YouTube videos on scabies in children: A cross-sectional study

**DOI:** 10.1371/journal.pone.0310508

**Published:** 2024-10-17

**Authors:** Emine Ozdemir Kacer, Ilayda Kacer

**Affiliations:** 1 Faculty of Medicine, Department of Pediatrics, Aksaray University, Aksaray, Turkey; 2 Faculty of Medicine, Department of Dermatology, Eskisehir Osmangazi University, Eskisehir, Turkey; Ufuk University Faculty of Medicine: Ufuk Universitesi Tip Fakultesi, TÜRKIYE

## Abstract

**Background:**

Recently, there has been an increase in scabies cases among young children in low- and middle-income countries. With the rise of online health information, platforms such as YouTube have become popular sources of disease-related content, but the accuracy of this information remains a concern.

**Aim:**

This study evaluates the reliability and quality of YouTube videos concerning scabies in children to address the lack of research in this area.

**Materials and methods:**

A cross-sectional analysis was conducted on April 1, 2024, reviewing the first 200 relevant YouTube videos with the search terms “scabies” and “scabies in children.” Videos were assessed using modified DISCERN (mDISCERN), Global Quality Score (GQS), and Journal of the American Medical Association (JAMA) scoring systems. Statistical analysis included descriptive statistics, Kruskal-Wallis tests, and Spearman correlation analysis.

**Results:**

Out of 200 videos, 107 met the inclusion criteria. The average mDISCERN score was 2.17, GQS was 2.63, and JAMA was 2.05, indicating generally poor quality. Videos by patients had the highest quality scores, while those from academic institutions had the highest JAMA scores. Longer videos with higher view counts were associated with better quality.

**Conclusion:**

This study reveals that the majority of YouTube videos on scabies in children are of low quality. There is a need for healthcare professionals to produce more accurate and reliable content to improve the quality of information available on YouTube. Further research should focus on enhancing the quality of health information on digital platforms.

## Introduction

Scabies is a prevalent and highly transmissible skin condition caused by the ectoparasitic mite, *Sarcoptes scabiei* (S. scabiei) [1]. Its global prevalence varies significantly, with reported rates ranging from 0.3% to 71% [2,3]. Due to the mite’s ability to spread through both direct and indirect contact, multiple individuals within a family can be simultaneously affected [4,5]. The intense itching, particularly severe during nighttime, substantially disrupts sleep, daily activities, and overall quality of life. The resultant scratching can lead to secondary bacterial infections such as pyoderma, impetigo, and cellulitis [6]. Treating scabies is complex, necessitating that all close contacts undergo treatment concurrently, even if they do not exhibit symptoms, to prevent the spread and recurrence of the infestation [7].

Recent years have witnessed a marked increase in scabies cases, largely driven by factors such as mass human migration, unchecked population growth, and limited access to healthcare [8]. This condition disproportionately affects young children, especially those aged between 1 and 4 years, in low- and middle-income countries [1,9].

As online platforms for health information have gained popularity, more patients are turning to these resources for information on diseases and their treatment options [10]. YouTube, a widely used video-sharing platform, is frequently accessed by both patients and healthcare providers for medical information [11]. Studies have shown that many individuals use YouTube to update their knowledge and gather information before seeking medical care [12,13]. However, as a public platform where anyone can upload content, there is a risk of disseminating inaccurate or misleading health information [14]. Therefore, it is critical to evaluate the quality, relevance, and timeliness of the medical content available on YouTube.

Recently, there has been an upsurge in studies exploring the role of social media in the dissemination of health information. Numerous studies have assessed the accuracy and quality of medical information in YouTube videos, covering topics such as rheumatoid arthritis [15], heart failure [5], hypertension [6], and the COVID-19 pandemic [7]. However, to our knowledge, no research has specifically focused on evaluating the reliability and quality of YouTube videos about scabies in children. This study aims to fill that gap by conducting a comprehensive analysis of the reliability and quality of YouTube videos concerning scabies in children.

## Materials and methods

### Study design and ethics

This study analyzed publicly available YouTube videos, without involving human participants or animals, thereby not requiring ethical approval, consistent with other similar YouTube-based studies.

### Data collection

A cross-sectional study was conducted on April 1, 2024, to assess the reliability and quality of YouTube videos containing the search terms "scabies" and "scabies in children." To eliminate potential biases in video recommendations based on previous viewing history, the search history was cleared before conducting the search. The first 200 videos were selected based on relevance as ranked by YouTube’s algorithm. Videos that were duplicates, not in English, lacked audio, or were advertisements were excluded. For videos consisting of multiple parts or episodes, they were treated as a single entry for this study.

After determining the suitability of the videos, they were evaluated by a dermatologist (I.K.) and a pediatrician (E.O.K.). In cases where there was a discrepancy between the ratings given by the two researchers, a third independent researcher (I.K.) was consulted for a final assessment.

### Video parameters

The study evaluated various characteristics of the videos, including upload date, video duration, uploader identity, number of views, likes, dislikes, and engagement index [(number of likes-number of dislikes) / total number of views × 100%]. The uploaders were categorized into five groups: physicians, patients, health-related websites, academic institutions/universities, and news agencies.

### Educational quality and reliability of the videos

Three distinct scoring systems—modified DISCERN (mDISCERN), Global Quality Score (GQS), and Journal of the American Medical Association (JAMA)—were employed to assess video quality and reliability [16–18]. Each video was independently evaluated using these scoring systems.

The mDISCERN tool utilizes a 5-point scale to measure the credibility and quality of a video. Points are awarded if the video is concise, credible, consistent, properly referenced, and effectively addresses any uncertainties. A higher score indicates greater credibility [16]. The mDISCERN scale is shown in Table 1.

**Table 1:** Modified DISCERN scale.

Score	Description
1	Are the video’s aims clear, concise, and achieved?
2	Are valid and reliable sources cited?
3	Is the information discussed balanced and unbiased?
4	Are additional sources of information listed for patient reference?
5	Does the video address areas of controversy and uncertainty?

Each “yes” to a question adds 1 point to the final score.

The GQS uses a five-point Likert scale to rate the overall quality of the videos [17]. Scores range from one to five, with five representing the highest quality and one the lowest. The results are categorized into low quality (1–2 points), medium quality (3 points), and high quality (4–5 points). The Global Quality Scale is shown in Table 2.

**Table 2:** Global quality scale.

Score	Description
1	Poor quality, poor flow, most information missing, not useful for patients.
2	Generally poor quality and flow, some information provided but of limited use to patients.
3	Moderate quality, suboptimal flow, some information is adequately discussed, somewhat useful for patients.
4	Good quality and flow, most relevant information is discussed, useful for patients.
5	Excellent quality and flow, very useful for patients.

The JAMA score assesses the accuracy and reliability of videos, evaluating four criteria: authority, quality, clarity, and timeliness. Each criterion is rated on a scale of one to four, with higher scores indicating greater accuracy [18]. The JAMA scale is shown in Table 3.

**Table 3:** JAMA scale.

Score	Description
1	Authorship: are author/contributor credentials and their affiliations provided?
2	Attribution: is copyright information listed, and references/sources for content provided?
3	Currency: is the initial date of posted content and dates of subsequent updates to content provided?
4	Disclosure: are conflicts of interest, funding, sponsorship, advertising, support, and video ownership fully disclosed?

Each “yes” to a question adds 1 point to the final score.

JAMA, Journal of the American Medical Association.

### Statistical analysis

Statistical analyses were conducted using the Statistical Package for the Social Sciences (SPSS) 25.0, developed by IBM Corp. The Shapiro-Wilk test was used to check for normality of data distribution. Descriptive statistical methods, including frequency, percentage, mean, and standard deviation, were used to analyze demographic data. The Kruskal-Wallis test was employed to compare quantitative data across groups, with post hoc analysis performed using the Bonferroni correction. The Bonferroni correction adjusts probability (p) values because of the increased risk of a type I error when making multiple statistical tests [19]. Categorical data were compared using the chi-square test, and Spearman correlation analysis was conducted to examine relationships between quantitative variables. Inter-rater agreement was assessed using the Kappa coefficient. Results were evaluated at a 95% confidence interval with a p-value less than 0.05 considered significant, and a p-value of less than 0.017 was set as the threshold for significance for the Bonferroni correction (0.05/3).

## Results

**Fig 1:**
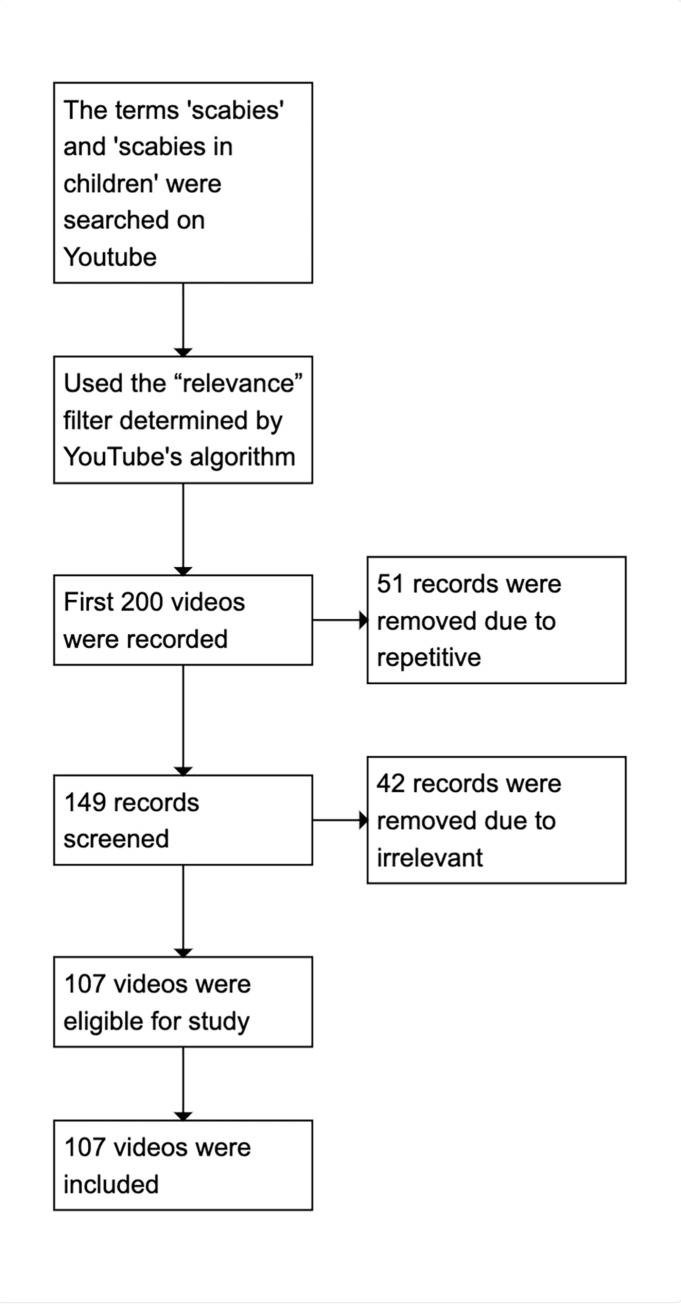
The study’s flow diagram.

After excluding 42 irrelevant and 51 repetitive videos, 107 videos were included for evaluation. The study’s flow diagram is shown in Fig 1. The most common types of uploaders were academic institutions or university hospitals, health-related websites, and patients. The content primarily consisted of general information about scabies, information specific to scabies in children, and patient experiences. The average DISCERN score was 2.17 out of 5, the average GQS score was 2.63 out of 5, and the average JAMA score was 2.05 out of 4. According to the GQS, 54.2% of the videos were of low quality, 29.9% were of medium quality, and 15.8% were of high quality. Table 4 presents the features, quality, and reliability scores of the videos.

**Table 4:** Features, quality, and reliability scores of videos.

**Source of upload**	**N**	**%**
Physician	3	2.8
Patient	13	12.1
Health-related websites	29	27.1
Academic/University	54	50.4
News agency	8	7.5
**Video content** General explanation of scabies	**N** 49	**%** 45.8
Scabies in children	31	28.9
Symptoms of scabies	2	1.9
Treatment of scabies	4	3.7
Patient experience	21	19.6
**Video features** Duration (minutes)	**Mean ±SD** 18.72 ± 31.45	**Min-max** (1–145)
Time since upload (months)	40.36 ± 34.66	(0–127)
Number of views	10,082.55 ± 56,015.90	(7–341,522)
Number of likes	338.91 ± 1970.62	(1–12,000)
Number of dislikes	0.66 ± 2.52	(0–16)
Interaction index	98.66 ± 3.91	(80–100)
mDISCERN	2.17 ± 1.01	(1–5)
JAMA	2.05 ± 1.03	(1–4)
Global Quality Scale	2.63 ± 0.88	(1–5)
**Global Quality Scale**	**N**	**%**
Low	58	54.2
Medium	32	29.9
High	17	15.8

JAMA, Journal of the American Medical Association. mDISCERN, Modified DISCERN.

Videos uploaded by patients had the longest average duration (27.11 ± 23.45) and the highest GQS and mDISCERN scores, while those uploaded by academic institutions/university hospitals had the highest JAMA scores. Table 5 details the duration, quality, and reliability scores of the videos according to the uploader category.

**Table 5:** Video duration, quality, and reliability scores for each uploader.

	Duration of video		GQS		JAMA		mDISCERN	
	Mean ±SD	Min-max	Mean ±SD	Min-max	Mean ±SD	Min-max	Mean ±SD	Min-max
Physician	18.27 ± 11.23	(1–48)	2.5 ± 0.46	(1–5)	2.5 ± 0.52	(1–4)	2.6 ± 0.46	(1–5)
Patient	27.11 ± 23.45	(1–132)	2.9 ± 0.57	(1–5)	2.6 ± .0.7	(1–4)	3 ± 0.92	(1–5)
Health-related websites	12.01 ± 46.32	(1–103)	2.5 ± 0.83	(1–5)	2.4 ± 0.69	(1–4)	2.6 ± 0.52	(1–5)
Academic/University	8.98 ± 56.76	(1–145)	2.4 ± 0.97	(1–5)	2.7 ± 0.89	(1–4)	2.55 ± 0.89	(1–5)
News agency	3.42 ± 7.14	(1–27)	2 ± 0.57	(1–3)	2 ± 0.51	(1–3)	2 ± 0.12	(1–3)

mDISCERN, Modified DISCERN.

In terms of quality classification, academic institutions/university hospitals were the most frequent uploaders of videos in all three groups. Most videos from health-related websites, news agencies, and academic institutions/university hospitals were of low quality, while videos uploaded by patients were generally of medium quality. Significant differences were observed in mDISCERN scores, JAMA scores, and average video duration. Pairwise comparisons revealed statistically significant differences between mDISCERN and JAMA scores in all post hoc comparisons. A significant difference was also found in the average video length between the low-quality and high-quality groups. Table 6 presents a comparison of video features based on quality classification.

**Table 6:** Comparison of video features according to quality classification.

	Low quality		Medium quality		High quality		*p*
	N	%	N	%	N	%	0.044^a^
Physician	1	33.3	1	33.3	1	33.3	
Patient	1	7.6	10	76.9	2	15.4	
Health-related websites	15	51.7	8	27.5	6	20.7	
Academic/University	34	62.9	12	22.2	8	14.8	
News agency	7	87.5	1	12.5	0	0	
	**Median (min–max)**		**Median (min–max)**		**Median (min–max)**		
mDISCERN	1 (1–2)		2 (1–4)		4 (2–5)		**< 0.001** ^b^ ^*^
JAMA	1 (1–3)		2 (1–4)		3 (2–4)		**< 0.001** ^b^ ^*^
Duration of video	3 (1–9)		4 (1–145)		54 (5–62)		**< 0.001** ^b^ ^**^
Number of views	328 (32–4230)		641 (7–3486)		678 (76–341,536)		0.063^b^
Number of likes	4 (1–16)		5 (1–178)		9(1–12,000)		0.038 ^b^
Number of dislikes	0 (0–1)		0 (0–3)		0 (0–16)		0.223 ^b^
Interaction index	100 (90.90–100)		100 (80.4–100)		100 (99.86–100)		0.416 ^b^

^a^Chi-square test

^b^Kruskal-Wallis test.

*Bonferroni test, significance between low and medium quality, low and high quality, medium and high quality *p* < 0.017.

**Bonferroni test, significance between low and high quality *p* < 0.017.

mDISCERN, Modified DISCERN.

A notable positive correlation was found between mDISCERN, GQS, and JAMA scores and both the number of views and video duration. However, no significant correlation was observed between the mDISCERN, JAMA, and GQS scores and the Engagement Index. Additionally, a significant positive correlation was observed among the three quality and reliability measures. Table 7 presents the correlation between video features and video classification scales.

**Table 7:** Correlation of video features with video classification scales.

		mDISCERN	JAMA	GQS
Number of views	r	.274	.308	.329
Duration of video	p r	0.05* .668	0.027* .566	0.017* .559
Interaction index	p r	< 0.001** -.073	< 0.001** .008	< 0.001** -.07
	p	0.662	0.956	0.67
mDISCERN	r	-	.673	.721
JAMA	p r	- .677	< 0.001** -	**< 0.001** **
Global Quality Scale	p r	< 0.001** .721	- .755	< 0.001** -
	p	**< 0.001** **	**< 0.001** **	-

*Correlation is significant at the 0.05 level.

**Correlation is significant at the 0.01 level.

JAMA, Journal of the American Medical Association. mDISCERN, Modified DISCERN. GQS, Global Quality Scale.

## Discussion

The global increase in scabies incidence, driven by multiple factors, alongside the widespread practice of seeking information online, highlights the importance of video content on platforms like YouTube. To our knowledge, this study is the first to evaluate the quality of scabies-related videos on YouTube. Contrary to findings from previous studies [20,21], our research indicates that although academic institutions and university hospitals uploaded more videos than other groups, the majority of these were of lower quality. Furthermore, our study found that videos with higher view counts and longer durations were associated with higher quality, reliability, and accuracy scores.

YouTube is widely utilized by both patients and healthcare professionals. Despite the advantages of accessing disease and treatment information on YouTube, prior studies have highlighted the variable quality of these videos [11]. One study analyzing YouTube videos found that their quality was generally moderate [10]. However, a review of similar studies on different diseases revealed that the overall quality was often low [22,23] Our findings align with this, showing that most of the analyzed videos were of suboptimal quality. Among the videos classified as high, medium, and low quality, the majority were uploaded by academic institutions and university hospitals, with low-quality videos often being promotional in nature.

The aim of this study was to perform a thorough quality assessment of YouTube videos addressing scabies in children. The Interaction Index was utilized as a measure to gauge the popularity of these videos. The decision to use the Interaction Index alongside viewing metrics (such as views, likes, and dislikes) was made to minimize potential biases related to the upload date of the videos [24]. Previous studies have reported varying results regarding the relationship between likes, dislikes, views, and the quality and reliability of content [25–27]. However, in our study, we found no significant correlation between the Interaction Index and the JAMA, GQS, and mDISCERN scores of the videos. The underlying reasons for why video reliability and quality do not significantly influence the number of likes, dislikes, and views are not fully understood, but the existing literature explores the dynamics of social media platforms and algorithms from multiple angles [28–30].

Our findings indicated a significant positive relationship between mDISCERN, GQS, and JAMA scores and both the number of views and video length. While video length plays a crucial role in attracting viewers’ attention, it is important to note that this does not necessarily imply that the entire video is watched; viewers may only engage with portions of the content. Therefore, when designing video content and determining its duration, it is crucial to consider factors such as appropriate viewing time, the capacity of viewers to maintain attention throughout the video, and the quality of the information provided.

On the other hand, a growing body of research highlights the importance of patient storytelling as an effective tool for enhancing communication and understanding between patients. Sharing experiences related to disease progression, patient testimonies, and medical practices has been shown to positively impact both those who share their stories and the patients who listen to them [31]. In this context, it is suggested that despite many videos being of low quality, those featuring patients sharing their experiences may still be valuable for patients and their families.

Abed et al. found no significant correlation between video duration and quality [32]. Conversely, Hawryluk et al. reported that longer videos tended to be of higher quality [33]. In our study, we observed that videos of higher quality were generally longer and exhibited better reliability and accuracy scores. Despite YouTube algorithms favoring shorter videos that convey general information, longer videos featuring patient experiences were found to provide more specific and high-quality information.

### Limitations

This study has several limitations. Firstly, the sample size is relatively small, which may be due to the decreased number of video uploads during the pandemic period when research activities and content sharing were significantly reduced. Secondly, the analysis was limited to English-language videos, which constrains the generalizability of the findings. Previous studies have shown that online patient experiences can be biased by racial and socioeconomic disparities [34]. Thirdly, it is important to recognize that video rankings on YouTube can vary depending on the IP address and geographic location of the user conducting the search. Fourth, the evaluation was carried out with the awareness that metrics such as views, comments, and likes are subject to ongoing changes. Finally, while the modified DISCERN score has been utilized in numerous studies, the lack of a validation study for this specific scoring system constitutes a limitation of the current research.

## Conclusion

This study, the first of its kind, highlights the generally poor quality of scabies content for children on YouTube. Physicians, dermatologists, and healthcare organizations should be more proactive in generating accurate and reliable YouTube content about scabies. As the role of social media and online video in health education increases, the quality and reliability of this information must be carefully evaluated. Further research is needed to understand how to improve the quality of health information on platforms such as YouTube.
